# Olanzapine and peripheral metabolic dysregulation: organ-resolved mechanisms, risk, and MASLD-aligned care pathways

**DOI:** 10.3389/fphar.2025.1729264

**Published:** 2026-04-02

**Authors:** Shuwei Weng, Jinjxiu Lin, Dajun Chai

**Affiliations:** 1 Cardiovascular Department, The First Affiliated Hospital, Fujian Medical University, Key Laboratory of Metabolic Heart Disease in Fujian Province, Clinical Research Centre of Metabolic Cardiovascular Disease in Fujian Province, Fuzhou, China; 2 Cardiovascular Department, National Regional Medical Center, Binhai Branch of the First Affiliated Hospital, Fujian Medical University, Fuzhou, China

**Keywords:** olanzapine, peripheral metabolic dysregulation, patient monitoring, risk stratification, multi-organ mechanisms

## Abstract

This review examines how olanzapine drives metabolic injury beyond the brain and why an organ-resolved perspective is needed. We synthesize clinical signals of early weight gain, insulin resistance, dyslipidemia, and steatotic liver disease, and integrate translational evidence across liver, adipose tissue, skeletal muscle, pancreatic β-cells, and the gut–liver axis. Mechanistic strands include disordered hepatic lipid handling, suppression of brown-fat thermogenesis, β-cell endoplasmic-reticulum stress with impaired secretion, and skeletal-muscle insulin-signaling defects with altered epigenetic programs that blunt glucose disposal. We summarize modifiers of risk across life stage, treatment exposure, genetic variation, smoking status, and pregnancy, and distill a pragmatic pathway that prioritizes early reassessment, MASLD-aligned liver evaluation, targeted lifestyle treatment, metformin for early deterioration, and GLP-1 receptor agonists when required. We advance the view that weight-independent extra-cerebral mechanisms are central to olanzapine’s metabolic liability and that psychiatric practice should adopt metabolic frameworks used in hepatology and endocrinology. We propose an agenda for organ-specific human phenotyping and exposure-aware designs that integrate therapeutic drug monitoring with microbiome, metabolomics, and bile-acid profiling, alongside comparative trials that test stepped algorithms within psychiatric care. This perspective outlines a path to preserve antipsychotic efficacy while reducing preventable systemic metabolic harm.

## Hightlights

1. Summarizes clinical and experimental evidence showing that olanzapine causes early, organ-specific metabolic disturbances beyond central appetite effects.
2. Outlines multiple peripheral mechanisms across the liver, adipose tissue, skeletal muscle, pancreas, and gut‐liver axis that jointly drive metabolic injury.
3. Integrates MASLD-aligned liver risk evaluation into psychiatric metabolic monitoring to improve early detection and intervention.
4. Proposes a practical care pathway combining structured monitoring, lifestyle therapy, and mechanism-based use of metformin or GLP-1 receptor agonists.

## 1 Introduction

Mental disorders impose a substantial and persistent global burden. Depressive disorders account for a large share of years lived with disability across regions, and schizophrenia likewise contributes markedly to worldwide morbidity and disability, as delineated by the Global Burden of Disease 2019 analyses (GBD, 2019 Mental Disorders Collaborators, 2022; Solmi et al., 2023). These data underscore the scale of illness that modern psychopharmacology must address.

Within this clinical landscape, olanzapine is widely prescribed as a second-generation antipsychotic for schizophrenia and bipolar disorder across acute and maintenance phases, and it is also used as an adjunct in major depressive disorder. Its broad adoption reflects robust antipsychotic efficacy and clinically meaningful mood-stabilizing properties that translate into symptom control and relapse prevention in routine care (Kolli et al., 2023). At the same time, comparative syntheses consistently associate olanzapine with prominent extra-cerebral metabolic adverse effects among second-generation antipsychotics, including weight gain, insulin resistance, dyslipidemia, steatotic liver disease, and increased risks of type 2 diabetes and cardiovascular disease. These metabolic signals are reproducible across designs and settings, with quantitative evidence for atherogenic lipid changes complementing broader cardiometabolic risk profiles (Carli et al., 2021; Mortimer et al., 2023; Li et al., 2020).

Current perspectives on mechanism remain unsettled. Traditional explanations emphasize centrally mediated changes in appetite and energy balance that secondarily drive weight gain and downstream metabolic stress. However, converging mechanistic and translational evidence indicates that direct peripheral effects in liver, adipose tissue, skeletal muscle, pancreatic β-cells, and other metabolic organs can rapidly perturb fuel handling after olanzapine exposure, in some cases before overt weight gain. Olanzapine-related metabolic injury has historically been framed as a downstream consequence of centrally mediated appetite change, positive energy balance, and eventual weight gain (Kroeze et al., 2003; Reynolds and Kirk, 2010). Emerging translational and controlled human data challenge that single-pathway view. Hepatocytes exposed to olanzapine upregulate lipogenic programs, accumulate triglyceride, and develop steatotic stress that is consistent with Metabolic dysfunction–Associated Steatotic Liver Disease (MASLD) injury (Shamshoum et al., 2021; Li et al., 2021; Albaugh et al., 2011; Lauressergues et al., 2010); adipose depots show suppressed thermogenesis and preferential lipid storage despite controlled intake (Shamshoum et al., 2021; Albaugh et al., 2011); peripheral insulin resistance and exaggerated postprandial insulin secretion can be induced within days in carefully phenotyped volunteers and experimental models, even before clinically meaningful weight gain (Shamshoum et al., 2021; Chintoh et al., 2008; Teff et al., 2013; Toledo et al., 2022). These findings indicate that liver, adipose tissue, skeletal muscle, and pancreatic β-cells exhibit drug-responsive metabolic vulnerability that does not depend on prolonged hyperphagia or obesity. This helps explain why hepatic fat accumulation, adverse lipid remodeling, and glycemic instability may emerge early in treatment, in some cases before overt obesity, and it argues that weight tracking alone is insufficient: organ-level metabolic surveillance is biologically justified.

Against this backdrop, we provide a narrative synthesis that moves beyond the brain to focus on peripheral metabolic dysregulation associated with olanzapine. We summarize recent clinical and translational research, highlight areas of consensus and contention regarding central versus peripheral drivers, and integrate these insights with contemporary metabolic nomenclature and practice, including the metabolic dysfunction–associated steatotic liver disease framework. The goal is to inform future investigation and to outline cross-disciplinary, organ-informed care pathways that preserve psychiatric stability while addressing systemic metabolic harm.

## 2 Clinical landscape of olanzapine-associated peripheral metabolic outcomes

Signals from translational and clinical research increasingly align to show that olanzapine exposure tracks with extra-cerebral metabolic phenotypes, particularly along pathways that culminate in steatotic liver disease and organ-level dysregulation (Li et al., 2021). Randomized effectiveness data from CATIE demonstrated greater weight gain and larger adverse shifts in fasting glucose and lipid measures with olanzapine than with several comparators, reinforcing its high-liability status among second-generation antipsychotics (Li et al., 2021). Head-to-head meta-analytic comparisons similarly place olanzapine near the top for weight gain and for increases in total cholesterol relative to other agents (Galling et al., 2016). Quantitative synthesis focused on lipid endpoints confirms atherogenic change during olanzapine treatment in routine care, adding granularity to the broader cardiometabolic risk profile (Li et al., 2020).

Evidence for downstream disease extends beyond composite metabolic-syndrome indices. Adult observational cohorts associate olanzapine use with higher incidence of type 2 diabetes, and the signal remains after adjustment for clinical covariates (Rummel-Kluge et al., 2010). Pediatric and adolescent data point in the same direction: a systematic review and meta-analysis found increased cumulative risk of type 2 diabetes in antipsychotic-exposed youth compared with psychiatric controls, with olanzapine prescription and longer exposure contributing to risk accrual over time (Leslie and Rosenheck, 2004). Early-phase studies in first-episode, drug-naïve patients documented rapid weight gain and lipid perturbations within weeks of initiation, indicating that surveillance should begin at treatment onset rather than after substantial weight change has occurred (Sengupta et al., 2005).

Liver-directed concerns are now part of the clinical picture. Critical and clinical reviews link atypical antipsychotic exposure—including olanzapine—to steatotic liver disease via convergent effects on insulin sensitivity, lipid handling, and inflammatory signaling (Xu and Zhuang, 2019; Gangopadhyay et al., 2022). These observations resonate with reports of higher NAFLD/MASLD burden in schizophrenia populations and with antipsychotic use as a correlate of liver fat accumulation and injury. Although attribution to a single drug can be complicated by lifestyle and comorbidity, the population-level, clinical, and mechanistic strands of evidence consistently argue for integrating liver assessment into antipsychotic safety workflows.

Variation across patients shapes the magnitude and tempo of metabolic change. Larger absolute shifts are repeatedly observed in antipsychotic-naïve or early-phase patients, and youth appear particularly susceptible to progressive dysglycemia on longer follow-up (Leslie and Rosenheck, 2004; Sengupta et al., 2005). Data directly contrasting oral versus long-acting olanzapine on metabolic outcomes remain limited and mixed across agents, so changing formulation should not be assumed to mitigate risk without targeted evidence. Concomitant mood stabilizers are common; while average pharmacokinetic interactions with valproate appear modest, randomized trials in acute mania report greater weight gain with olanzapine-based regimens than with divalproex, suggesting that regimen composition and cumulative exposure shape the expressed phenotype in practice (Tohen et al., 2002; Spina et al., 2009).

These clinical observations motivate a pragmatic monitoring agenda. Clinicians should anticipate early weight gain and adverse lipid shifts, expect glycemic risk to accumulate over time—especially in younger and antipsychotic-naïve patients—and incorporate liver-focused evaluation aligned with contemporary steatotic liver disease frameworks. This landscape provides the empirical scaffold for the mechanistic and management sections that follow.

## 3 Peripheral organ mechanisms of olanzapine-associated metabolic injury

A growing body of translational evidence indicates that olanzapine exerts direct extra-cerebral actions on metabolic organs. Acute and subacute *in vivo* studies show rapid disturbances of fuel handling across the liver, adipose tissue, pancreatic β-cells, and the gut–liver interface, providing biologically coherent pathways that align with the clinical dysmetabolism summarized previously (Shamshoum et al., 2021). Critically, several of these disturbances emerge within days to weeks of exposure at clinically relevant doses and plasma concentrations. They have been demonstrated even in weight-stable healthy volunteers and in acute rodent models. Short-term olanzapine can induce peripheral insulin resistance with exaggerated insulin and C-peptide responses, suppress skeletal-muscle glucose disposal, activate hepatocellular lipogenesis, and blunt brown adipose thermogenesis before overt weight gain develops. These findings indicate that the metabolic liability is closely linked to exposure itself rather than requiring prolonged hyperphagia or chronic adiposity, and that early biochemical alterations can precede visible anthropometric change (Albaugh et al., 2011; Lauressergues et al., 2010; Chintoh et al., 2008; Teff et al., 2013; Toledo et al., 2022).

Weight-independent hepatic injury has been reproduced in mice and linked to dysregulated triglyceride trafficking via apolipoprotein A5 and sortilin, with concordant rises in plasma triglycerides and reduced circulating apoA5 after short-term exposure, and with hepatocyte evidence of impaired intracellular sorting that favors intrahepatic lipid accumulation (Huang et al., 2022). Independent human–animal data further implicate endoplasmic-reticulum stress, where activation of the PERK–eIF2α–ATF4–CHOP axis accompanies olanzapine-related dyslipidemia and is detectable in patient samples as well as in experimental models, positioning ER-stress signaling as a driver rather than a bystander of hepatic dysmetabolism (Liu et al., 2024). Cholesterol homeostasis provides a second lever: in a randomized rat model of olanzapine-induced steatosis, metformin reversed hepatic fat and serum lipids while down-modulating LXRα and PCSK9 expression, arguing that sterol-sensing and PCSK9 pathways contribute to the steatotic phenotype and are pharmacologically tractable (Zhu et al., 2022). Earlier translational observations add reduced hepatic Akt phosphorylation and impaired insulin signaling to this picture, supporting a primary liver insulin-resistance component that can evolve before or independent of weight gain (Ren et al., 2019). Beyond lipid trafficking and ER-stress, dysregulation of Wnt/β-catenin signaling via its key effector TCF7L2 has been implicated in olanzapine-related metabolic injury. In preclinical models, perturbing TCF7L2 exacerbates weight gain and insulin resistance under olanzapine exposure, linking canonical Wnt signaling to downstream hepatic and peripheral insulin action and positioning TCF7L2 as a candidate node for organ-resolved intervention (Li et al., 2018). Together with the apoA5–sortilin axis, these strands place ER-stress, insulin-signaling defects, and LXRα/PCSK9-linked cholesterol handling as complementary mechanisms converging on MASLD-like injury under olanzapine exposure.

Direct interference with muscle insulin signaling has been shown *in vitro* and *in vivo*. In L6 myotubes, clinically relevant olanzapine concentrations suppress insulin-stimulated glucose uptake and key signaling intermediates, indicating muscle-intrinsic insulin resistance (Engl et al., 2005). Short clinical exposures in healthy volunteers produce measurable epigenetic remodeling of insulin-signaling genes in skeletal muscle, including large methylation shifts at PPARGC1A, consistent with rapid transcriptional re-programming that could impair oxidative metabolism and glucose disposal (Burghardt et al., 2024). Obesity magnifies these acute defects: in a double-blind crossover study, a single olanzapine dose elicited greater insulin resistance and ectopic lipid signals in individuals with obesity than in normal-weight controls, highlighting how host phenotype gates the metabolic expression of the drug (Townsend et al., 2018). Given the established cross-talk between TCF7L2 activity and insulin signaling in muscle and adipose depots, the TCF7L2 axis provides a mechanistic bridge that may integrate olanzapine-induced defects in glucose uptake with adipose storage dynamics (Li et al., 2018).

Adipose tissue contributes through reduced thermogenic capacity and increased storage. Rodent studies demonstrate suppression of brown adipose tissue thermogenesis during olanzapine treatment, including reductions in UCP1-linked heat production and brown fat temperature under pair-feeding conditions; locomotor activity falls but does not fully account for the energy imbalance (Zhang et al., 2014; Stefanidis et al., 2009). Pharmacologic modulation such as simvastatin can partially restore brown adipose activity in young rats, reinforcing a causal role for thermogenic impairment (Liu et al., 2020). Complementary data indicate downregulation of UCP1-dependent thermogenesis alongside the development of insulin resistance, further supporting an adipose-centric mechanism beyond hyperphagia alone (Wang et al., 2022). In combination, lowered energy expenditure and increased white-fat storage are expected to amplify lipid overflow to the liver and skeletal muscle.

Pancreatic β-cells show vulnerability to olanzapine-induced stress. At clinically relevant micromolar concentrations, olanzapine activates endoplasmic reticulum stress pathways—including PERK/eIF2α and IRE1/XBP1—in insulin-secreting cells, impairs glucose-stimulated insulin secretion, and allows functional rescue with tauroursodeoxycholic acid *in vitro* (Grajales et al., 2022). Independent experiments link olanzapine to β-cell apoptosis through disruption of PERK-mediated translational attenuation, providing a mechanistic bridge from cellular stress to secretory failure (Ozasa et al., 2013). These findings position β-cell dysfunction as a potential early event that can precede or evolve independently of overt weight change.

Interactions along the gut–liver axis further modulate exposure and toxicity. Olanzapine alters the gut microbiome, and microbial depletion increases systemic bioavailability by approximately 1.8-fold in rats while shifting intestinal xenobiotic-metabolizing enzymes such as UGT1A3 (Cussotto et al., 2021). Antibiotic attenuation of olanzapine-induced metabolic dysfunction in rodents supports a contributory role for the microbiota in shaping both pharmacokinetics and host metabolic responses (Davey et al., 2013). These bidirectional influences make a case for gastrointestinal and hepatic surveillance when antipsychotics are initiated or escalated. Complementing pharmacokinetic and microbial-depletion data, targeted microbiome modulation with Akkermansia muciniphila ameliorated olanzapine-induced MASLD *in vivo*, accompanied by hepatic PGRMC1/SIRT1/FOXO1 pathway activation, supporting gut-directed strategies to mitigate liver injury under antipsychotic exposure (Chen et al., 2025).

**FIGURE 1:**
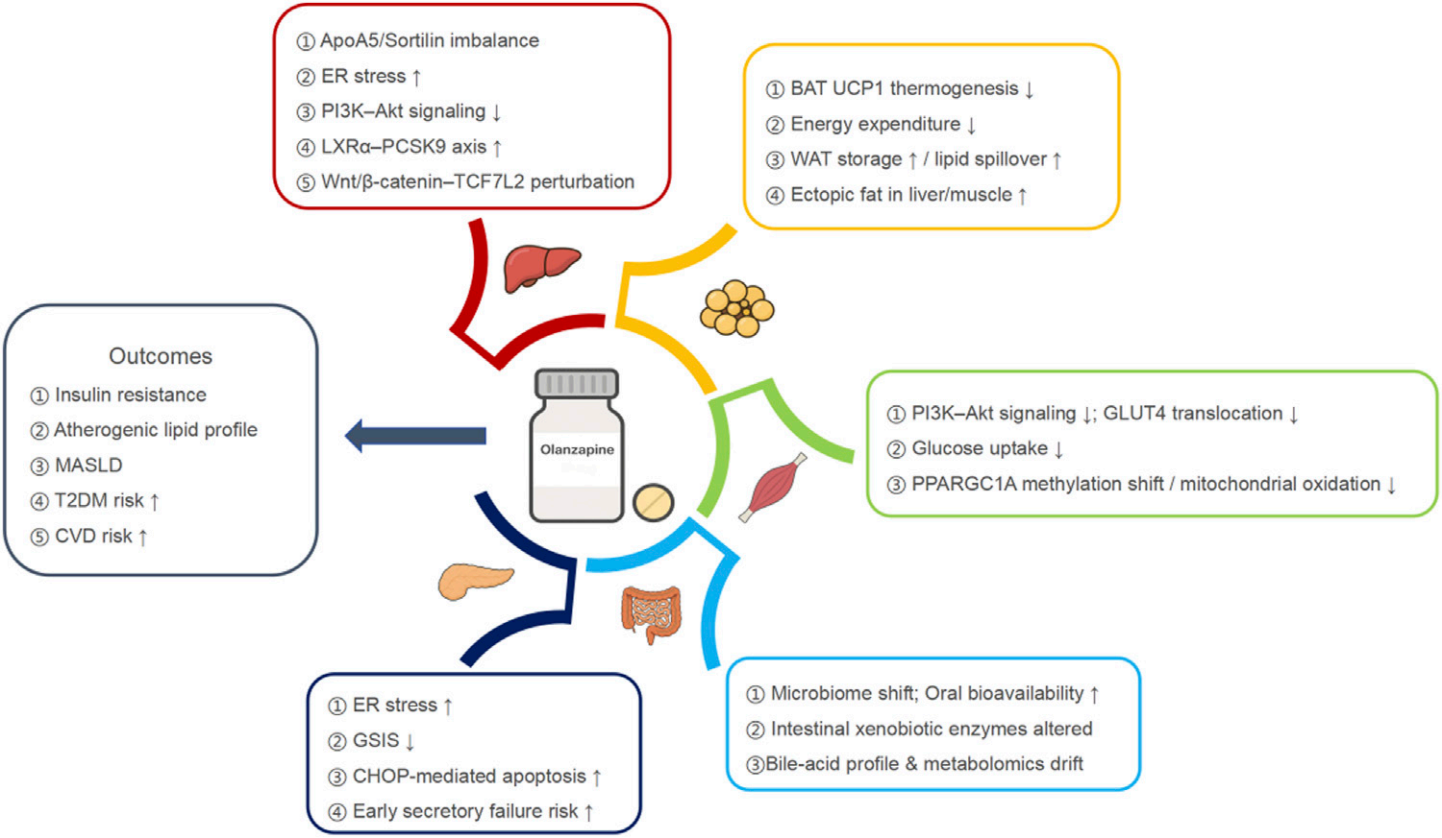
Organ-resolved model of olanzapine-associated peripheral metabolic injury and actionable checkpoints.

Olanzapine-induced metabolic injury is not a set of isolated toxicities confined to single organs; instead it appears as a repeatable pattern of early stress across multiple peripheral metabolic tissues. The liver shows SREBP-driven *de novo* lipogenesis, triglyceride accumulation, and steatotic stress consistent with a MASLD-like phenotype (Lauressergues et al., 2010; Chintoh et al., 2008). Adipose depots fail to buffer nutrient load efficiently: oxidative and thermogenic capacity fall, brown fat output is suppressed, and lipid is preferentially stored rather than oxidized, lowering whole-body energy expenditure and increasing lipid spillover toward ectopic sites (Zhang et al., 2014; Stefanidis et al., 2009; Liu et al., 2020; Wang et al., 2022). Skeletal muscle, the dominant site of insulin-stimulated glucose disposal, demonstrates reduced glucose uptake and impaired oxidative metabolism, favoring systemic insulin resistance and ectopic lipid deposition (Engl et al., 2005; Burghardt et al., 2024; Townsend et al., 2018). Pancreatic β-cells attempt to compensate for peripheral insulin resistance with sustained hypersecretion, but that compensatory drive is itself stressful; endoplasmic-reticulum stress pathways are activated, proinsulin handling is impaired, and secretory reserve narrows (Grajales et al., 2022; Ozasa et al., 2013). Across these tissues, unresolved ER stress recurs as a shared feature: in hepatocytes it amplifies lipogenesis and inflammatory signaling; in adipose and muscle it interferes with insulin signaling; and in β-cells it produces secretory strain and vulnerability to failure (Engl et al., 2005; Burghardt et al., 2024; Townsend et al., 2018; Zhang et al., 2014; Stefanidis et al., 2009; Liu et al., 2020; Wang et al., 2022; Grajales et al., 2022; Ozasa et al., 2013). The combined effect is progressive insulin resistance, dyslipidemia, and steatotic liver injury that can become clinically evident early, in some cases before overt obesity is established. This supports the view that olanzapine’s metabolic liability is fundamentally extra-cerebral and multi-organ, rather than a simple downstream consequence of weight gain, and it underlies Figure 1.

## 4 Risk modifiers and susceptible populations

Clinically meaningful heterogeneity is the rule rather than the exception with olanzapine-associated dysmetabolism. Children and adolescents display larger short-term metabolic shifts than adults when second-generation antipsychotics are initiated, and in a randomized, treatment-naïve pediatric cohort olanzapine produced the greatest early increases in weight and lipids. These early changes set the trajectory for downstream risk and justify intensified surveillance from the very start of treatment in younger patients. First-episode and antipsychotic-naïve adults show a similar pattern, with steeper early weight and lipid changes soon after olanzapine initiation, reinforcing the need to front-load monitoring in the first weeks rather than waiting for visible adiposity to accumulate (Correll et al., 2009).

Inter-individual biology further modifies risk beyond age and illness stage. Pharmacogenetic syntheses point to common variation in serotonergic signaling as a contributor to antipsychotic-induced weight gain, with replicated protective effects of the HTR2C −759T allele on weight trajectories during second-generation antipsychotic exposure. The original discovery linking weight gain to a 5-HT2C receptor promoter polymorphism provides a mechanistic foothold in appetite and energy-balance pathways and supports a precision-risk perspective in patients who show rapid early gain or report strong family histories of metabolic disease (Lett et al., 2012; Reynolds et al., 2002).

Exposure and drug–environment interactions also shape phenotype in practice. Cigarette smoking induces CYP1A2 and lowers olanzapine concentrations at a given dose, whereas smoking cessation increases exposure; therapeutic drug-monitoring data demonstrate substantially higher steady-state serum levels in non-smokers than in smokers at comparable doses. Because higher exposure can amplify both efficacy and metabolic adverse effects, major changes in smoking status should prompt dose review and closer biochemical surveillance. Concomitant mood stabilizers are common; although average pharmacokinetic interactions with valproate are modest, randomized trials in acute mania report greater weight gain with olanzapine-based regimens than with divalproex alone, consistent with an additive metabolic burden when olanzapine anchors the regimen. These observations argue for regimen-level risk accounting rather than single-drug thinking (Tohen et al., 2002; Spina et al., 2009; Laika et al., 2010).

Pregnancy introduces a physiologically insulin-resistant milieu in which antipsychotic exposure has measurable metabolic consequences. Population-based analyses identify olanzapine, clozapine, and quetiapine use during pregnancy as associated with higher risk of gestational diabetes after adjustment for psychiatric and obstetric covariates, underscoring the need for anticipatory glycemic surveillance when these agents are required for maternal stability (Heinonen et al., 2022). Broader registry data corroborate an overall increase in gestational diabetes among antipsychotic-treated pregnancies, further supporting a proactive monitoring stance in this setting (Bodén et al., 2012). Beyond maternal glycemic control, pregnancy also creates a second at-risk patient: the fetus. Olanzapine-class agents cross the placenta, and perinatal registry data link *in utero* exposure to both higher maternal gestational diabetes rates and downstream offspring signals such as increased large-for-gestational-age birth and upward shifts in birthweight distribution (Heinonen et al., 2022; Bodén et al., 2012). These neonatal phenotypes are metabolically relevant because excessive fetal growth in a hyperglycemic, hyperlipidemic intrauterine environment is widely regarded as an early marker of overnutrition and future insulin resistance. Conceptually, this positions pregnancy not only as a period in which maternal hepatic fat, β-cell workload, and insulin resistance are amplified, but also as a developmental window in which the fetal liver, pancreatic β-cell mass, adipose storage capacity, and hypothalamic appetite-set circuitry may be durably programmed toward greater lipogenesis and impaired glucose handling. Direct, organ-resolved longitudinal evidence in humans is still limited, but the convergence of heightened gestational diabetes risk in the mother and fetal overgrowth signals in the offspring argues that perinatal monitoring should be framed as dual-patient metabolic surveillance, with planned pediatric follow-up rather than viewing pregnancy risk solely through the lens of short-term maternal glucose (Heinonen et al., 2022; Bodén et al., 2012).

Pre-existing cardiometabolic burden appears to amplify the magnitude of deterioration once olanzapine is introduced. Patients with obesity or dyslipidemia more commonly encountered in severe mental illness exhibit larger absolute shifts when a drug with an atherogenic lipid signature is selected, a pattern consistent with quantitative syntheses of olanzapine’s effects on triglycerides and cholesterol (Li et al., 2020). Links between atypical antipsychotics and steatotic liver disease provide a parallel rationale for liver-directed assessment when baseline fatty liver is present or suspected, aligning clinical observation with mechanistic accounts of insulin sensitivity, lipid handling, and inflammatory signaling (Xu and Zhuang, 2019; Gangopadhyay et al., 2022).

Translating these modifiers into practice favors an explicit risk-stratification step before or at initiation. Younger or antipsychotic-naïve patients who tend to show rapid early gain, individuals with pharmacogenetic or family-history markers of dysmetabolism, non-smokers or recent quitters at standard olanzapine doses with higher expected exposure, patients receiving olanzapine-anchored polypharmacy for mania, pregnant patients or those planning conception, and those with pre-existing dyslipidemia or steatotic liver disease should be considered higher risk and prioritized for front-loaded surveillance. In these groups, early laboratory reassessment within the first one to 3 months complements weight and waist tracking and increases the chance of detecting weight-independent hepatic or glycemic injury that would otherwise be missed (Correll et al., 2009; Lett et al., 2012; Reynolds et al., 2002; Laika et al., 2010; Heinonen et al., 2022; Bodén et al., 2012).

## 5 Monitoring and mitigation: From early detection to targeted intervention

Routine, structured monitoring is the linchpin for minimizing extra-cerebral harm without compromising psychiatric stability. Consensus guidance developed by psychiatric and diabetes organizations recommends baseline assessment before or at initiation, followed by early re-checks in the first 1–3 months and sustained surveillance thereafter. Core elements include weight and waist, blood pressure, fasting plasma glucose or HbA1c, fasting lipids, and liver enzymes; documentation of personal/family cardiometabolic history and tobacco use helps anticipate exposure and risk (American Diabetes Association and American Psychiatric AssociationAmerican Psychiatric Associa tionAmerican Association of Clinical EndocrinologistsNorth American Association for the Study of Obesity, 2004). Health-system programs show that embedding these checkpoints into clinic workflows increases screening rates, although adherence wanes without deliberate quality-improvement cycles (Soda et al., 2021). Practical pharmacy-led algorithms translate the consensus into concrete schedules and action thresholds that can be implemented in psychiatric settings (DeJongh, 2021).

Lifestyle modification retains first-line status and is effective in serious mental illness when delivered with sufficient intensity. A structured group behavioral weight-loss program for outpatients with schizophrenia, schizoaffective disorder, or bipolar disorder produced clinically meaningful reductions in weight and waist circumference over 18 months, with concomitant improvements in cardiometabolic markers, demonstrating that tailored behavioral treatment is feasible and effective in this population (Daumit et al., 2013).

Adjunctive pharmacotherapy is justified when early monitoring detects rapid weight gain, dyslipidemia, or dysglycemia despite behavioral steps, or when baseline risk is high. Metformin has the strongest evidence base: meta-analysis supports attenuation of olanzapine-associated weight gain and improvement in insulin sensitivity measures, with benefits apparent within the first months of treatment (de Silva et al., 2016). For patients with established weight gain and impaired glucose tolerance during clozapine or olanzapine therapy, a randomized, placebo-controlled trial of the GLP-1 receptor agonist liraglutide improved glucose tolerance, reduced body weight, and lowered waist circumference and fasting insulin, pointing to a mechanism-concordant option when metformin is inadequate or contraindicated (Larsen et al., 2017). Real-world series suggest semaglutide may benefit antipsychotic-associated weight gain refractory to metformin, though controlled data are still emerging and careful coordination with psychiatry is needed to preserve symptom control (Prasad et al., 2023).

Choice and dose of antipsychotic remain relevant levers when clinical status allows. Comparative trials and meta-analyses consistently place olanzapine among the highest-liability agents for weight and lipids relative to several alternatives (Galling et al., 2016); where psychiatric efficacy can be maintained, selecting or transitioning to a lower-liability option may be preferable. Regardless of agent, changes in smoking status alter olanzapine exposure via CYP1A2; therapeutic drug-monitoring data show higher steady-state concentrations in non-smokers than smokers at the same dose, so cessation or heavy-smoking reduction should prompt dose review and closer metabolic follow-up (Laika et al., 2010).

Hepatic surveillance should be aligned with contemporary steatotic liver disease practice. Hepatology guidelines endorse non-invasive, stepwise risk stratification using routine labs and ultrasound where indicated, reserving elastography or specialist referral for patients with biochemical abnormalities, imaging evidence of steatosis with metabolic risk, or indeterminate fibrosis scores (European Association for the Study of the Liver EASLEuropean Association for the Study of Diabetes EASDEuropean Association for the Study of Obesity EASO, 2016). Recent consensus on the renaming and definition of metabolic dysfunction–associated steatotic liver disease (MASLD) establishes positive metabolic criteria and standardized terminology for steatotic liver disease (Rinella et al., 2023). Given evidence linking atypical antipsychotics, including olanzapine, with steatotic liver disease and broader metabolic injury, liver risk assessment in antipsychotic-treated patients should follow MASLD-aligned, non-invasive pathways. In practice, abnormal transaminases, persistent hypertriglyceridemia, or weight-independent dysglycemia after olanzapine initiation should trigger liver-directed evaluation and consideration of GLP-1–based or metformin therapy alongside lifestyle measures.

Putting these elements together favors a staged pathway: obtain baseline metrics; re-check weight, glucose, lipids, and ALT/AST at 1–3 months; escalate monitoring frequency in high-risk groups defined as described above; implement structured lifestyle treatment incorporating Mediterranean-style or low–glycemic-index dietary patterns rich in unsaturated fats, fiber, and whole grains to reduce steatotic and cardiometabolic stress, together with supervised aerobic and resistance exercise programs that have demonstrated feasibility and benefit in serious mental illness populations (Daumit et al., 2013; Ryan et al., 2013; Vancampfort et al., 2017); initiate lifestyle treatment early; add metformin when rapid gain or glycemic worsening is detected; consider GLP-1 receptor agonists when glucose intolerance or obesity persists despite metformin; and reassess antipsychotic selection and dosing if psychiatric stability can be preserved with a lower-liability alternative. This approach operationalizes the mechanistic and epidemiologic signals into a workflow that aims to maintain symptom control while reducing long-term cardiometabolic and hepatic harm. In higher-risk patients, this workflow should increasingly be applied in an organ-specific way: liver-directed follow-up aligned with MASLD criteria when transaminases, triglycerides, or weight-independent dysglycemia worsen (Xu and Zhuang, 2019; Gangopadhyay et al., 2022; European Association for the Study of the Liver EASLEuropean Association for the Study of Diabetes EASDEuropean Association for the Study of Obesity EASO, 2016; Rinella et al., 2023), early β-cell stress assessment when exaggerated insulin secretion or postprandial hyperinsulinemia appears (Teff et al., 2013; Toledo et al., 2022; Grajales et al., 2022; Ozasa et al., 2013), and targeted support of adipose thermogenesis and energy expenditure in patients who show rapid fall in activity and early gain despite minimal intake change (Zhang et al., 2014; Stefanidis et al., 2009; Liu et al., 2020; Wang et al., 2022). Beyond currently implemented measures, translational work also points to microbiome-directed strategies. Experimental manipulation of the gut microbiota has been shown to blunt olanzapine-associated weight gain, insulin resistance, adipose inflammatory activation, and ectopic lipid deposition, indicating that the gut microbial community is not merely a marker of metabolic liability but a modifiable driver of it. In preclinical models, targeted restoration of beneficial taxa such as Akkermansia muciniphila has been reported to attenuate olanzapine-induced insulin resistance and steatotic, MASLD-like liver injury through defined gut–liver signaling pathways, suggesting a potential hepatometabolic mitigation approach that extends beyond metformin and GLP-1 receptor agonists (Davey et al., 2013; Chen et al., 2025; Qian et al., 2023). These microbiome-focused approaches remain investigational and are not yet established in prospective clinical trials, but they illustrate how organ-level biology can generate future mechanism-based adjuncts to standard metabolic monitoring.

## 6 Outlook: Advancing research on Olanzapine’s extra-cerebral effects

Future work needs to move beyond composite metabolic endpoints toward organ-resolved human biology that can separate central from peripheral drivers and convert mechanisms into testable interventions. Prospective cohorts should synchronize anthropometry, fasting lipids, postprandial glycemic testing, and liver-directed metrics such as transaminases, ultrasound when indicated, and fibrosis scores. This design would clarify the ordering of dyslipidemia, dysglycemia, and steatosis under olanzapine exposure and build directly on randomized effectiveness trials, head-to-head syntheses, and quantitative lipid meta-analyses (Li et al., 2020; Lieberman et al., 2005; Galling et al., 2016). Early scheduling within the first weeks to months is essential because controlled human experiments and preclinical liver studies already show weight-independent insulin resistance and steatosis as early events, and rodent data point to rapid changes in hepatic and adipose physiology (Zhang et al., 2014; Stefanidis et al., 2009; Liu et al., 2020; Wang et al., 2022). Liver endpoints and terminology should follow non-invasive, stepwise pathways from hepatology guidance and the MASLD consensus, so that psychiatric services speak the same language as liver clinics (Soda et al., 2021; DeJongh, 2021). For the gut–liver axis, trials should evaluate live biotherapeutics such as Akkermansia muciniphila alongside dietetic programs, with MASLD-aligned non-invasive liver endpoints and mechanistic readouts in the PGRMC1/SIRT1/FOXO1 pathway (Chen et al., 2025).

Interventional studies should align with the implicated peripheral organs rather than treat metabolic injury as a single undifferentiated construct. For hepatic triglyceride handling, trials should test whether metformin or GLP-1 receptor agonists accelerate normalization of enzymes and imaging markers in patients receiving olanzapine, using MASLD-aligned non-invasive readouts as primary outcomes (de Silva et al., 2016; Larsen et al., 2017; Prasad et al., 2023; European Association for the Study of the Liver EASLEuropean Association for the Study of Diabetes EASDEuropean Association for the Study of Obesity EASO, 2016; Rinella et al., 2023). For adipose thermogenesis, protocols that pair energy-expenditure phenotyping with structured lifestyle programs and, when needed, pharmacologic add-ons can quantify restoration of UCP1-linked function and downstream insulin sensitivity (Zhang et al., 2014; Stefanidis et al., 2009; Liu et al., 2020; Wang et al., 2022). For signaling-targeted approaches, studies that stratify by Wnt/β-catenin–TCF7L2 activity and test pathway-modulating strategies could clarify whether TCF7L2 status predicts organ-specific response (Li et al., 2018). For β-cell stress, small mechanistic trials that track postprandial insulin secretion can determine whether agents with ER-stress-modulating activity reverse early secretory defects in patients who develop dysglycemia during olanzapine treatment (Grajales et al., 2022; Ozasa et al., 2013).

Exposure biology should be measured directly, not inferred. Microbiome–pharmacokinetic work shows that bacterial depletion increases olanzapine bioavailability and that shifts in gut communities alter xenobiotic-metabolizing enzymes (Cussotto et al., 2021; Davey et al., 2013). Future cohorts should integrate a tri-omics exposure framework that combines therapeutic drug monitoring with microbiome, metabolomics, and bile acid profiling, and should account for smoking status, which changes olanzapine concentrations through CYP1A2 induction. The goal is to test whether exposure differences map to organ-specific injury and whether dose adjustments, timing strategies, or targeted adjuncts reduce that injury while preserving antipsychotic efficacy (Laika et al., 2010).

Precision-risk strategies require oversampling and prespecified analyses in vulnerable groups. Children and adolescents show larger early metabolic shifts, first-episode or antipsychotic-naïve adults deteriorate quickly after initiation, pharmacogenetic variation at HTR2C modulates weight trajectories, and pregnancy adds an insulin-resistant physiological state with higher gestational diabetes risk when certain antipsychotics are used (Correll et al., 2009; Lett et al., 2012; Reynolds et al., 2002; Laika et al., 2010; Heinonen et al., 2022; Bodén et al., 2012). Risk tools developed in such enriched cohorts should be validated against hard outcomes such as discontinuation for metabolic harm, incident diabetes, or incident steatotic liver disease rather than intermediate markers alone.

Mitigation algorithms should be tested head to head with durability and safety endpoints in psychiatric settings. Lifestyle programs tailored to serious mental illness are feasible and effective, metformin attenuates olanzapine-associated weight gain, liraglutide improves glucose tolerance and body weight in clozapine- or olanzapine-treated patients, and emerging real-world signals suggest semaglutide benefits cases that do not respond to metformin (Daumit et al., 2013; de Silva et al., 2016; Larsen et al., 2017; Prasad et al., 2023). Pragmatic trials that randomize to metformin-first versus GLP-1-first strategies, with predefined switches for non-response and careful tracking of psychiatric stability, would clarify sequencing, generalizability, and long-term safety.

The agenda outlined above is constrained by several factors that qualify interpretation and generalizability. A substantial share of mechanistic data comes from animal models and brief human experiments, while sex and age distributions are often unbalanced and pediatric and pregnant populations are underrepresented. Many cohorts are small with short follow up, and observational designs remain vulnerable to residual confounding and indication bias. Organ resolved intermediate endpoints are not consistently linked to hard outcomes such as incident diabetes, clinically adjudicated MASLD, or treatment discontinuation for metabolic harm. Therapeutic drug monitoring and tri-omics exposure panels—microbiome, metabolomics, and bile acid profiling—and advanced liver imaging are unevenly available across settings, which limits exposure-aware designs and hinders replication. Evidence for GLP-1 receptor agonists beyond single randomized trials and case series is still maturing, and applicability across psychiatric indications and treatment stages requires confirmation. These constraints should guide cautious inference and motivate multicenter studies that align organ specific endpoints with patient relevant outcomes.

## 7 Conclusion

Olanzapine’s metabolic harm extends beyond centrally mediated appetite to organ-level disturbances across liver, adipose tissue, skeletal muscle, and pancreatic β-cells, with clinical signals spanning early weight gain, atherogenic dyslipidemia, progressive glycemic risk, and steatotic liver disease. A practical path forward is clear: front-load monitoring in high-risk groups, integrate MASLD-aligned liver assessment into routine care, and deploy mechanism-concordant mitigation beginning with intensive lifestyle treatment, metformin when early deterioration appears, and GLP-1 receptor agonists when needed, while reconsidering antipsychotic selection if stability allows. Psychiatry should begin to manage olanzapine the way hepatology and endocrinology manage MASLD and prediabetes: not as ‘weight gain,’ but as a definable pattern of hepatic fat stress, β-cell overwork, suppressed adipose thermogenesis, and skeletal-muscle insulin resistance that can be followed and acted on as discrete, clinically actionable targets. Future work should anchor trials in organ-resolved endpoints, measure exposure biology directly, and develop validated risk tools so that psychiatric efficacy is preserved without accepting preventable systemic metabolic injury.
